# A Joint Parallel Timing Recovery Loop with Low Complexity for Terahertz Communication System and Its FPGA Implementation

**DOI:** 10.3390/s26041163

**Published:** 2026-02-11

**Authors:** Feifei Wang, Wentao Wang, Linshan Xue, Xianggang Liu, Huichao Zhou

**Affiliations:** China Academy of Space Technology, Beijing 100094, China; doublefeif@163.com (F.W.); xuelinshanx@alu.uestc.edu.cn (L.X.);

**Keywords:** terahertz communication, timing recovery loop, timing error detector, FPGA implementation

## Abstract

This paper proposes a low-complexity joint parallel timing recovery loop, which is well-suited for large-bandwidth terahertz (THz) communication systems. Specifically, the loop is jointly composed of a modified matched filter (MMF) and a timing error detector (TED), where sampling point offset correction is achieved by deleting, holding, or retaining data in parallel data caches (DCs), and timing phase error compensation is implemented by sliding the coefficients of the MMF. The feasibility of the proposed loop is verified using both Gardner and O&M TED. Numerical simulation results demonstrate that the loop operates efficiently, with a performance loss of less than 0.1 dB compared to the theoretical bit error rate (BER) curve. Furthermore, the loop is implemented on a THz field-programmable gate array (FPGA) platform, successfully realizing parallel demodulation of 15 Gbps 64QAM high-speed signals at 220 GHz. Notably, the proposed loop effectively reduces hardware resource consumption under a parallel architecture, providing a viable solution to address the current shortage of on-board resources in high-speed THz communication systems.

## 1. Introduction

With the rapid development in communication technology, people put forward higher requirements for information transmission rate. However, the current communication systems cannot meet the increasing demand for high-speed data transmission. Terahertz communication has the advantages of abundant spectral resources, large capacity, high energy efficiency, and large transmission bandwidth. Using the terahertz frequency band for high-speed communication can efficiently alleviate the situation of increasingly strained spectrum resources and break through the capacity limitation of the current wireless communication systems, which is an important technical path for the future development in wireless communication. So we take terahertz communication as the background to expand this paper.

There have been numerous studies focusing on high-speed wireless communication systems in the terahertz band, with breakthroughs spanning transmission performance [1,2,3,4,5,6]. Meanwhile, the modem will directly determine the performance of the communication systems. At present, the modulation mode of high-speed data transmission system is mostly MPSK, which is relatively simple and mature. With the increase of transmission rate and shortage of spectrum resources, the problem of low transmission efficiency caused by low-order modulation has become increasingly severe, failing to meet practical demands. Therefore, higher-order modulation methods (MQAM, MAPSK, etc.) begin to be used in high-speed modems [7,8]. However, high-order modulation remains a major challenge in terahertz (THz) communication systems. Its implementation involves higher complexity and reduces the Euclidean distance between adjacent constellation points, rendering the system extremely susceptible to timing errors. Even a tiny deviation from the optimal sampling point can induce intersymbol interference (ISI). Moreover, the stronger phase fluctuations and path loss inherent in the THz band further amplify this effect, thereby imposing much more stringent requirements on the synchronization performance of the communication systems.

In the actual communication systems, transceiver transmission delays prevent receivers from sampling at each symbol’s optimal point, introducing timing errors that degrade demodulation performance, especially for high-order modulated terahertz systems. Therefore, timing recovery plays a crucial role in the overall performance of the receiver. Some mainstream timing error detection algorithms have been proposed in [9,10,11,12]. In 1978, Mueller and Muller (M&M) first proposed a timing error detection algorithm that each symbol only needs one sampling point, but it is decision-directed and affected by frequency offset [9]. In 1980, L.E. Franks proposed a timing error detection algorithm based on the maximum-likelihood estimation theory [10]. In order to solve the problem of carrier frequency offset influence and high complexity, F. M. Gardner proposed a timing error detection algorithm in 1986, which only needs two sampling points for each symbol, and was widely used in all-digital communication systems [11]. In 1993, F. M. Gardner extended his algorithm [13], and proposed a feedback architecture of all-digital timing recovery loop based on Farrow interpolation algorithm [14]. In 1988, M. Oerder and H. Meyr proposed a timing error detection algorithm with better performance for higher-order modulation, and the timing error estimate is unbiased [12]. Both feedforward [15] and feedback architectures are used in timing recovery loop. Feedforward enables fast acquisition but has low synchronization accuracy, suiting burst communications; feedback requires longer capture/convergence time but delivers high accuracy, needing only error magnitude/direction guidance for adjustment and thus being widely adopted in long-term continuous systems [16,17,18,19,20,21]. With the rapid development in high-speed wireless communication, Traditional serial timing recovery schemes are constrained by logic device clock frequencies in high-speed wireless systems, driving demand for parallel timing synchronization. Numerous timing recovery architectures applicable to various usage scenarios have been proposed [5,6,15,22,23,24,25,26]. Parallel Gardner-based via interpolation filters were proposed in [22,23,25,26,27], while C. Lin designed a frequency-domain parallel architecture based on O&M TED with a dual-feedback loop, which is firstly reported in Alternate Parallel Receiver (APRX) [24,28,29].

Amid the rapid development in reconfigurable hardware platforms and the increasing trend toward full digitization of receiver algorithms, hardware resource constraints remain a critical challenge for FPGA implementations in large-bandwidth terahertz communication systems. Numerous efforts have been devoted to reducing FPGA resource consumption of various receiver modules, timing recovery still stands out as a resource bottleneck  [5,30,31,32]. In this paper, we propose a low-complexity joint parallel timing recovery loop based on a feedback time-domain architecture, which innovatively integrates timing error compensation with MMF and TED to jointly accomplish efficient timing recovery. In the proposed design, sampling point offset correction is realized by deleting, holding, or retaining data in parallel DCs, while timing phase error compensation is achieved by sliding/adjusting the MMF coefficients directly in the time domain, eliminating the need for resource-intensive FFT/IFFT operations and significantly reducing hardware resource consumption. And we use high-order modulation to improve spectral efficiency and use parallel polyphase filter technique to save resources.

The rest of this paper is organized as follows: in Section 2, the *N* parallel time-domain timing recovery system architecture is given. Section 3 gives the specific working details of the timing recovery module. In Section 4, the methods of timing sampling point offset correction and timing phase error compensation in time domain are proposed. Then the simulation and implementation results are given in Section 5. Finally, we draw conclusions in Section 6.

## 2. Parallel Timing Recovery System Model

**Figure 1:**
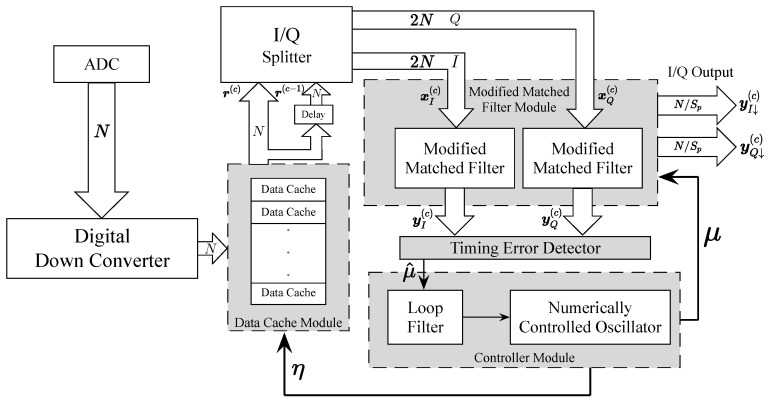
The *N* parallel time-domain feedback timing recovery loop architecture.

The joint *N* parallel paths timing recovery loop architecture is shown in Figure 1; all modules within the feedback loop contribute to the timing recovery process. The loop is mainly composed of four modules: data caches (DCs) module, modified matched filter (MMF) module, timing error detector (TED) module and controller module. Different from the traditional timing recovery loop, the loop proposed in this paper combines matched filter and timing error detector to jointly complete the timing recovery by shifting the matched filter coefficients to compensate the timing errors. This loop adopts high-order modulation to improve frequency band utilization and can be used in high-speed communication systems.

Firstly, a time-continuous intermediate frequency (IF) signal r(t) is received. The received signal can be written as

(1) r(t)=Re[u(t)]cos2πfct+Im[u(t)]sin2πfct=I(t)cos2πfct+Q(t)sin2πfct,

where $f_{c}$ is the carrier frequency of the IF signal, I(t) and Q(t) represent the in-phase and quadrature-phase of the sent symbol respectively. And 

(2) $$u\left(t\right)=\sum_{n=-\infty }^{\infty }s_{n}g_{T}\left(t-nT-\tau \right),$$

where u(t) is the baseband equivalent complex envelope of the received modulated signal after down-conversion from the carrier frequency, $g_{T}\left(t\right)$ is the transmission signal pulse, *T* is symbol duration, $s_{n}$ are information symbols, and τ is an unknown, slowly varying timing delay that mainly caused by transmission delay and different sources of transceivers clocks. For simplicity, the effects of noise are not taken into account. In M-QAM modulation, $s_{n}=a_{n}+jb_{n}$ with $a_{n},b_{n}\in \left\{\pm 1,\pm 3,\cdots ,\pm \sqrt{M}-1\right\}$.

The received signal is input to the matched filter and gets the outputs

(3) $$\begin{array}{c} y\left(t\right)=r\left(t\right)*g_{R}\left(t\right), \end{array}$$

where ∗ represents the convolution operation and $g_{R}\left(t\right)$ is a receiving filter (impulse response).

**Figure 2:**
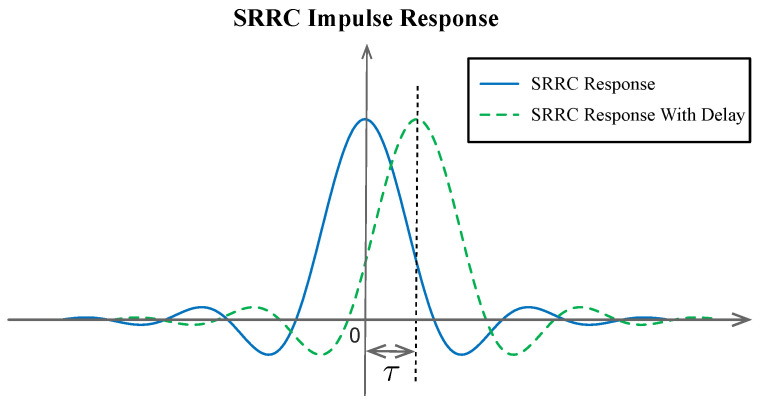
The impulse response of the SRRC filter (blue solid line). The filter has a delayed τ impulse response (green dashed line).

The timing error in the system is essentially caused by the mismatch between the transceiver sampling instants, which can be characterized by a time delay τ. The core goal of timing recovery is to compensate for this time delay to ensure optimal symbol sampling. To compensate for the timing delay τ, we need to obtain the filter output at the delayed instant (t+τ), i.e., y(t+τ). Based on the basic property of convolution, it can be expressed as follows

(4) $$\begin{array}{cc} y(t+\tau ) & =r\left(t\right)*g_{R}\left(t\right)*\Delta \left(t+\tau \right)\\ \; & =r\left(t+\tau \right)*g_{R}\left(t\right)\\ \; & =r\left(t\right)*g_{R}\left(t+\tau \right), \end{array}$$

where Δ(t) is the impulse function. From the analysis of (4), timing recovery can be expressed in two equivalent forms, which directly correspond to two timing error compensation methods. One realized by $r\left(t+\tau \right)*g_{R}\left(t\right)$, which requires shifting the input signal r(t) to align with the original matched filter $g_{R}\left(t\right)$. And another realized by $r\left(t\right)*g_{R}\left(t+\tau \right)$, which requires shifting the impulse response of the matched filter to $g_{R}\left(t+\tau \right)$. In practical digital communication systems, the received signal r(t) is sampled by the ADC, and it is difficult to flexibly implement time-domain sliding of the sampled signal. Therefore, we select the second method (sliding the matched filter) to compensate for the time delay τ. As illustrated in Figure 2, the time-domain shift of $g_{R}\left(t\right)$ to $g_{R}\left(t+\tau \right)$ does not alter the shape of the matched filter, only its time-domain position to adjusts the filter’s effective time delay to realize precise timing error compensation.

In practical communication systems, the accumulation of timing delay τ will cause optimal symbol slip at the receiver, resulting in the loss or repetition of symbols. Thus, the timing error compensation function of the recovery loop is divided into two core components: timing sampling point offset correction and timing phase error compensation.

The received signal r(t) firstly sampled by Analog to Digital Converter (ADC) at rate $f_{s}=1/T_{s}$, where $T_{s}$ is the sampling period. And $S_{p}$ represents the up-sampling ratio of the system. In line with Nyquist theorem, to ensure the sampled signal has no aliasing, choosing a suitable $S_{p}$ is very important. In this paper, to intuitively illustrate the I/Q separation principle and sequence mapping rule, we adopt a specific example assumption of $S_{p}=4$, $f_{s}=4f_{c}$. It should be emphasized that proposed scheme is compatible with any rational ratio of $f_{c}/f_{s}$ that satisfies the Nyquist sampling criterion. Thus, the received signal can be expressed in the in-phase and quadrature-phase (I/Q) form.

(5) $$\begin{array}{cc} r(mT_{s}) & =I\left(mT_{s}\right)\cos\left(2\pi \frac{f_{c}}{f_{s}}m\right)+Q\left(mT_{s}\right)\sin\left(2\pi \frac{f_{c}}{f_{s}}m\right)\\ \; & =I\left(mT_{s}\right)\cos\left(\frac{\pi }{2}m\right)+Q\left(mT_{s}\right)\sin\left(\frac{\pi }{2}m\right). \end{array}$$

Under this assumption, the received digital sequence can be expressed as

(6) $$\begin{array}{c} r=[I(0),Q(1),-I(2),-Q(3),I(4),Q(5)\ldots ]. \end{array}$$

The received signal sequence is parallelized into *N* parallel paths and written into *N* parallel data caches, and timing sampling point offset correction is achieved by controlling read operation of the data caches. For the convenience of operation, *N* is usually taken as an integer multiple of $S_{p}$ when designing the system. Reading the data $r^{\left(c\right)}$ from the data caches module at *c*-th clock, where $r^{\left(c\right)}$ is an N×1 vector of received sequence and *c* is an index of the clock signal.

In the actual communication system, the signal sequence is infinitely long, so it needs to be segmented to reduce system output delay and ensure the real-time performance of the system. There are two main methods of segmentation group convolution processing, including overlapping addition and overlapping reservation. To simplify the implementation, overlapping reservation is used in this loop. The last *N* sampling points spliced with the current *N* sampling points that read from the data cache module to form a new sequence, where the last *N* sampling points are placed at the front of the new sequence. After that, a new sequence $x^{\left(c\right)}={\left[r^{(c-1)T},r^{(c)T}\right]}^{T}$ is obtained, where $x^{\left(c\right)}$ is an 2N×1 vector and ${\left(\cdot \right)}^{T}$ represents the transpose of a vector. Sending $x^{\left(c\right)}$ to the I/Q splitter and get sequences $x_{I}^{\left(c\right)}$ and $x_{Q}^{\left(c\right)}$ respectively, where $x_{I}^{\left(c\right)}$ and $x_{Q}^{\left(c\right)}$ are L×1 vector, L=2N.

(7) $$\begin{array}{cc} x_{I}^{\left(c\right)} & =[I(0),0,-I(2),0,I(4)\ldots -I(L-2),0],\\ x_{Q}^{\left(c\right)} & =[0,Q(1),0,-Q(3),0,Q(5)\ldots -Q(L-1)]. \end{array}$$

Then I and Q-channel signal sequences are input to the MMF module separately to complete matched filter and timing phase error compensation by convolution. After the above operation, we discard the aliased data and get the output signal $y_{I}^{\left(c\right)}$ and $y_{Q}^{\left(c\right)}$, where $y_{I}^{\left(c\right)}$ and $y_{Q}^{\left(c\right)}$ are N×1 vector. Decimating $y_{I}^{\left(c\right)}$ and $y_{Q}^{\left(c\right)}$ at intervals $S_{p}$ to get loop output sequence $y_{I↓}^{\left(c\right)}$ and $y_{Q↓}^{\left(c\right)}$, which compose the output $y^{\left(c\right)}$ of the system.

(8) $$y^{\left(c\right)}=y_{I↓}^{\left(c\right)}+jy_{Q↓}^{\left(c\right)},$$

where $y_{I↓}^{\left(c\right)}$, $y_{Q↓}^{\left(c\right)}$ and $y^{\left(c\right)}$ are $N/S_{p}\times 1$ vector. $y^{\left(c\right)}$ is made up of the optimal sampling points of the system and all of which were sampled at the position where the signal SNR is maximized.

In MMF module, we introduce an index $\Theta_{tap}$ to control the matched filter coefficients sliding step to achieve timing phase error compensation. $y_{I}^{\left(c\right)}$ and $y_{Q}^{\left(c\right)}$ calculated by the MMF module are input to the TED module to calculate timing error estimate $\hat{\mu }$. Then input $\hat{\mu }$ to controller module to extract the timing phase out μ and the timing sampling offset indicator η. And the η extracted from the numerical control oscillator(NCO) is fed back to the data cache module. It controls the data caches control vector $r_{d}^{\left(c\right)}\in {\left\{0,1\right\}}^{N}$, which is used to achieve the timing sampling point offset correction. In order to compensate for the timing phase error, μ needs to be fed back to the MMF module to control the index $\Theta_{tap}$. These will be covered in detail in the next section.

## 3. Parallel Timing Recovery Loop

### 3.1. Modified Matched Filter

In communication systems, data transmission over a bandlimited channel requires pulse shaping to eliminate intersymbol interference (ISI). In bandlimited digital communication systems, using a pair of matched square-root-raised-cosine (SRRC) filters in transmitter and receiver which satisfies Nyquist first criterion, can achieve ISI-free theoretically. The delay τ between the transmitter and receiver needs to be compensated to recover the symbol information of the sender correctly. In this work, we propose a MMF, whose primary function is to implement timing phase error compensation.

Firstly, the received time-continuous, high-order QAM signal r(t) is sampled by ADC at rate $1/T_{s}$. Through parallel processing and the I/Q splitter, the received signal is separated into $x_{I}^{\left(c\right)}$ and $x_{Q}^{\left(c\right)}$. Due to the calculation of I and Q-channel is the same, for convenience, here we take I-channel as an example to analyze. Samples $x_{I}^{\left(c\right)}\left(mT_{s}\right)=x_{I}^{\left(c\right)}\left[m\right]$, m∈[(c−1)L,cL) are taken at uniform intervals $T_{s}$. Sending $x_{I}^{\left(c\right)}$ to the matched filter (MF), and we get the output. The process of matched filtering is actually to convolve the signal with $g_{R}\left(t\right)$, and then keep the correct convolution data to get a valid output $y_{I}\left(t\right)$,

(9) $$y_{I}\left(t\right)=\sum_{m}x_{I}^{\left(c\right)}\left(mT_{s}\right)g_{R}\left(t-mT_{s}\right)\;\left(N+1\right)T_{s}\leq t\leq 2NT_{s},$$

In the timing recovery loop, the timing error estimate $\hat{\mu }$ calculated by the TED is then sent to the controller module to get the appropriate parameter timing phase out μ. And then it is fed back to the MMF module to compensate the delay τ.

After compensation, the matched filter output $y_{I}\left(t\right)$ can be written as

(10) $$y_{I}\left(t+\mu \right)=\sum_{m}x_{I}^{\left(c\right)}\left(mT_{s}\right)g_{R}\left(t+\mu -mT_{s}\right).$$

In *N* parallel paths digital receiver, we propose a modified matched filter which is slidable. Thus, timing error compensation can be achieved by shifting $g_{R}\left(t\right)$. Resample $y_{I}\left(t+\mu \right)$ at time instants $t+\mu =kT_{i}$, where *k* is the index of the sequence calculated by the MMF module and $y_{I}^{\left(c\right)}\left(kT_{i}\right)=y_{I}^{\left(c\right)}\left[k\right]$, k∈[(c−1)N,cN) at the adjustable intervals $T_{i}$,

(11) $$y_{I}^{\left(c\right)}\left(kT_{i}\right)=\sum_{m}x_{I}^{\left(c\right)}\left(mT_{s}\right)g_{R}\left(kT_{i}-mT_{s}\right),$$

where *m* is signal index, we define the filter index *i* as follows

(12) $$\begin{array}{c} i=\left\lfloor kT_{i}/T_{s}\right\rfloor -m-\mu , \end{array}$$

where $\left\lfloor p\right\rfloor$ means largest integer not exceeding *p*. Also define the basepoint index $m_{k}$, which originates from the sampling index *m*, that is, $m_{k}=mT_{s}$ and $m_{k+1}=\left(m+S_{p}\right)T_{s}$. Between each two basepoint, there are $S_{p}-1$ samples. Thus, we get equation $\left(kT_{i}-mT_{s}\right)=\left(i+\mu \right)T_{s}$. The outputs of loop can be rewritten as

(13) $$\begin{array}{cc} y_{I}^{\left(c\right)}\left(kT_{i}\right) & =y_{I}^{\left(c\right)}\left[\left(m+\mu \right)T_{s}\right]\\ \; & =\sum_{i=0}^{N_{2}-1}x_{I}^{\left(c\right)}\left[kT_{i}-\left(i+\mu \right)T_{s}\right]g_{R}\left[\left(i+\mu \right)T_{s}\right]. \end{array}$$

In high-speed terahertz communication, the traditional serial architectures are no longer applicable, so the timing recovery loop proposed in this paper adopts *N*-path parallel architecture. Through the data cache module, the sequence $x_{I}^{\left(c\right)}$ is sent into the MMF module and convolved with the matched filter coefficients, which has excellent ISI rejection capability. After MMF module, we get the output sequence $y^{\left(c\right)}$ with adjustable interval $S_{p}T_{i}$. Also get the sequences $y_{I}^{\left(c\right)}$ and $y_{Q}^{\left(c\right)}$ that are input to TED module to calculate timing error estimate.

### 3.2. Controller

Controller module is indispensable in the timing recovery loop because the timing error estimate calculated by TED cannot be directly applied to the MMF module for compensation and the data cache module for correction. As is shown in Figure 1, the controller module is composed of loop filter (LF) and NCO in the timing recovery loop proposed in this paper. The role of the controller module is to extract the available information about basepoint index $m_{k}$ and timing phase out μ for timing sampling point offset correction and timing phase error compensation, respectively.

**Figure 3:**
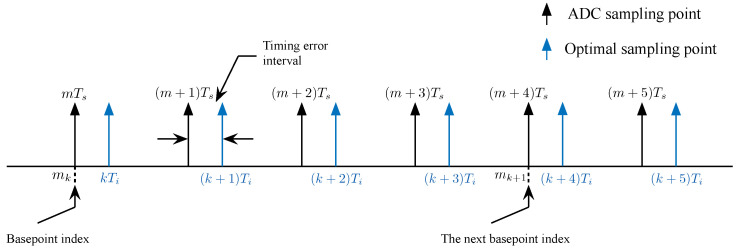
The relation between sampling points.

As is shown in Figure 3, the relation between ADC sampling points and optimal sampling points is illustrated. The sampling points are timed uniformly through at a fixed interval $T_{s}$ by ADC. Due to the different sources of transmitter and receiver clock, timing delay will inevitably occur in the system. With the accumulation of timing delay, the basepoint will be offset and $m_{k}$ is no longer the optimal basepoint index. Thus, we introduce a parameter η to represent the offset of the basepoint.

Under the *N*-path parallel architecture, the loop calculates the timing phase out every ρ symbols and $\rho =N/S_{p}$. Due to the slow change of timing error, sharing one μ among multiple sampling points will hardly introduce extra error. In parallel implementation, *N* sampling points are simultaneously input to the TED module to calculate the timing error estimate $\hat{\mu }$. Then send it to the LF to filter out the interference components mainly caused by noise and make the output smooth. The LF output $\mu_{l}\left(c\right)$ at the *c*-th clock is

(14) $$\begin{array}{cc} w_{1}\left(c\right) & =c_{1}\times \hat{\mu }\left(c\right),\\ w_{2}\left(c\right) & =c_{2}\times \hat{\mu }\left(c\right)+w_{2}\left(c-1\right),\\ \mu_{l}\left(c\right) & =w_{1}\left(c\right)+w_{2}\left(c\right), \end{array}$$

where $c_{1}$ and $c_{2}$ are LF coefficients, its design corresponds to the equivalent noise bandwidth of the loop [33]. In hardware implementation, the parameters are often set to integer multiples of 1/2, which facilitates replacing multiplication operations with shift operations.

**Figure 4:**
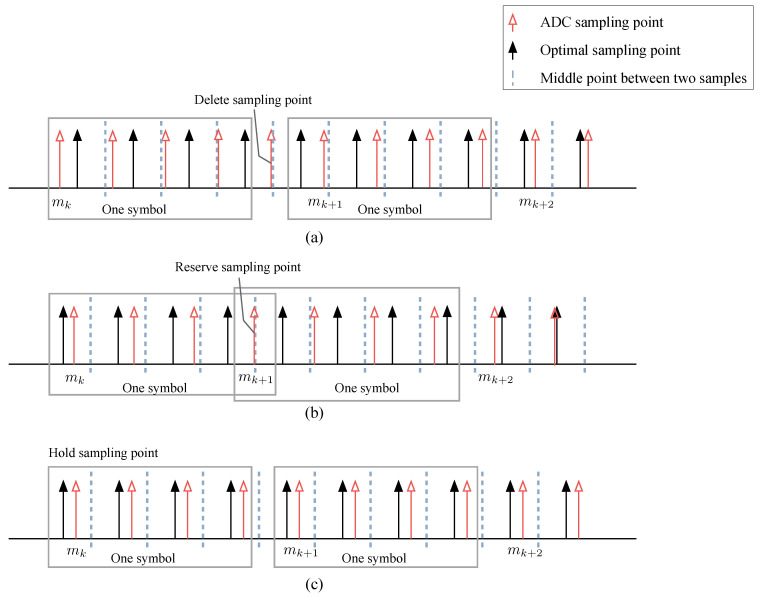
The operations of selecting basepoints for three different situations: (**a**) Delete. (**b**) Reserve. (**c**) Hold.

The smoothed timing error estimate $\mu_{l}$ is input to the NCO and used as the control word of the NCO. The difference equation can be expressed as

(15) $$\mu^{\prime }\left(c\right)=\mu \left(c-1\right)+\mu_{l}(c),$$

where $\mu^{\prime }$ is a temporary value of the timing phase out and μ is the timing phase out that extracts from the NCO. According to (15), it can be seen that the theoretical timing phase out will continuously increase or decrease to infinity. In order to limit the timing phase out to a fixed interval, the temporary value $\mu^{\prime }$ need to be recomputed to get the timing phase out μ, which is discussed as follow. According to the timing delay relation diagram between ADC sampling points and optimal sampling points given in Figure 4, we can see that with the continuous accumulation of timing delay, $\mu^{\prime }$ will exceed or lag one sampling point. And the current basepoint index is no longer suitable, so we use the parameter η to control the offset.

Figure 4a describes a situation that the ADC sampling rate is faster than the true symbol rate. As the result, the optimal sample point move forward as time pass and finally exceed the middle point of two adjacent ADC sampling points, thus, one ADC sampling point needs to be deleted (namely jumped over) and the basepoint moves one more point to the right side ($m_{k+1}=m_{k}+S_{p}+1$). In practical, η(c)=1 is fed back to the data cache module to achieve the deletion of the sampling point. In this situation, temporary value of the timing phase out $\mu^{\prime }$ is larger than 0.5 in value and it should be subtracted by 1 to get the timing phase out μ which is limited to a fixed interval (i.e., μ∈[−0.5,0.5)). Figure 4b describes an opposite situation that the ADC sampling rate is slower than the true symbol rate, and one sampling point needs to be reserved (i.e., η(c)=−1). Simultaneously, the basepoint moves one fewer point to the right side ($m_{k+1}=m_{k}+S_{p}-1$). Figure 4c demonstrates the situation that $\mu^{\prime }\in \left[-0.5,0.5\right)$, ADC sampling points do not need to be deleted or reserved (i.e., η(c)=0), it means the basepoint index $m_{k}$ has no more special operations to be done.

The timing recovery loop in this paper is a double feedback loop. After controller, we extract the timing phase out μ and the timing sampling point offset indicator η. μ is feedback to the MMF module to control the sliding step of the matched filter coefficients to achieve timing phase error compensation and η is feedback to the data cache module, which controls the reading of sampling points to delete, hold or reserve the ADC sampling points. After these two steps, timing error compensation at the receiver is completed. The controller algorithm for our timing recovery loop can be summarized as Algorithm 1:
**Algorithm 1** Controller algorithm for the timing recovery loop**Input:** The *c*-th clock timing error estimate $\hat{\mu }\left(c\right)$ from TED.  1: Input $\hat{\mu }\left(c\right)$ to the LF and get the smoothed timing error estimate $\mu_{l}$ according to (14).  2: $\mu^{\prime }\left(c\right)=\mu \left(c-1\right)+\mu_{l}\left(c\right)$.  3: **if** 
$\mu^{\prime }\left(c\right)>0.5$ **then**  4:        $\mu \left(c\right)=\mu^{\prime }\left(c\right)-1\;\eta \left(c\right)=1$  5: **else if** 
$\mu^{\prime }\left(c\right)\leq -0.5$ **then**  6:        $\mu \left(c\right)=\mu^{\prime }\left(c\right)+1\;\eta \left(c\right)=-1$  7: **else**  8:        $\mu \left(c\right)=\mu^{\prime }\left(c\right)\;\eta \left(c\right)=0$  9: **end if**  **Output:** Output the *c*-th time clock timing error phase out μ(c) and timing sampling point offset indicator η(c).

### 3.3. Timing Error Detector

The TED plays a crucial role on the system performance of the timing recovery loop. Gardner TED algorithm [11] and O&M TED algorithm [12] are enduring for a long time and have been widely used. The Gardner TED algorithm (taking QPSK as an example) can be expressed as

(16) $$\begin{array}{c} \hat{\mu }\left(c\right)=y_{I}^{\left(c\right)}\left(m_{k}-S_{p}/2\right)\left[y_{I}^{\left(c\right)}\left(m_{k}\right)-y_{I}^{\left(c\right)}\left(m_{k-1}\right)\right]\\ +y_{Q}^{\left(c\right)}\left(m_{k}-S_{p}/2\right)\left[y_{Q}^{\left(c\right)}\left(m_{k}\right)-y_{Q}^{\left(c\right)}(m_{k-1})\right]. \end{array}$$

In the case of high-order modulation, (16) needs to be modified. According to [34], we get the modified Gardner TED algorithm that suitable for high-order QAM modulation.

(17) $$\begin{array}{cc} \hat{\mu }\left(c\right) & =\left\{y_{I}^{\left(c\right)}\left(m_{k}-S_{p}/2\right)-\frac{y_{I}^{\left(c\right)}\left(m_{k}\right)+y_{I}^{\left(c\right)}\left(m_{k-1}\right)}{2}\right\}\\ & \times \left[y_{I}^{\left(c\right)}\left(m_{k}\right)-y_{I}^{\left(c\right)}\left(m_{k-1}\right)\right]\\ & +\left\{y_{Q}^{\left(c\right)}\left(m_{k}-S_{p}/2\right)-\frac{y_{Q}^{\left(c\right)}\left(m_{k}\right)+y_{Q}^{\left(c\right)}(m_{k-1})}{2}\right\}\\ & \times \left[y_{Q}^{\left(c\right)}\left(m_{k}\right)-y_{Q}^{\left(c\right)}(m_{k-1})\right]. \end{array}$$

O&M TED algorithm requires at least four samples for each symbol (e.g., $S_{p}\geq 4$), and the calculation is more complicated. The O&M TED algorithm can be expressed as

(18) $$\hat{\mu }\left(c\right)=-\frac{1}{2\pi }\arg\left(Y_{l}\left(c\right)\right),$$

where $Y_{l}\left(c\right)$ is the *l*-th data segment’s spectral components at *c*-th clock determined by computing the complex Fourier coefficient at the symbol rate. Every data segment includes EN samples, *E* is the cumulative length which means every data segment spans *E* clock.

(19) $$Y_{l}\left(c\right)=\sum_{n=l}^{E+l-1}Y\left(n\right).$$

Y(c) is the spectral components determined by *N* samples at *c*-th clock,

(20) $$Y\left(c\right)=\sum_{k=0}^{N-1}F^{\left(c\right)}\left[k\right]e^{-j2\pi k/S_{p}},$$

where $F^{\left(c\right)}\left[k\right]={\left(y_{I}^{\left(c\right)}\left[k\right]\right)}^{2}+{\left(y_{Q}^{\left(c\right)}\left[k\right]\right)}^{2}$.

The choice of the cumulative length *E* is related to the O&M TED’s estimate accuracy and the capture range of the timing phase error. So, choosing an appropriate cumulative length *E* is important.

## 4. Timing Recovery Loop Implementation

In the implementation of the *N*-path parallel timing recovery loop, how to achieve timing sampling point offset correction and timing phase error compensation is very important. The specific implementation details are as follows.

### 4.1. Modified Matched Filter Implementation

In the timing recovery loop, the timing phase out μ extracted from the controller is fed back to the MMF module, and the timing phase error is compensated by sliding the matched filter coefficients. In digital communication system, finite-impulse response (FIR) filter with SRRC characteristic is often used as matched filter. Generally, the filter is truncated into $S_{l}$ symbols, each symbol period contains $S_{p}$ samples. The order of filter is $S_{l}\times S_{p}$ and the length of its coefficients is $N_{2}=S_{l}\times S_{p}+1$. And the selection of the $S_{l}$ value is limited by the length of the signal sequence to ensure sufficient convolution. What’s more, the value of roll-off factor α determines the bandwidth of the signal that $B=\left(1+\alpha \right)R_{s}$, where $R_{s}$ is symbol rate, and we need to choose a suitable α value according to different TED application conditions. For example, Gardner TED is considered to be applicable for approximately 40–100 percent excess bandwidth of the signal [11]. However, higher α value means wider bandwidth the channel need, so choosing a proper α value is important to balance the resource of bandwidth and TED performance.

**Figure 5:**
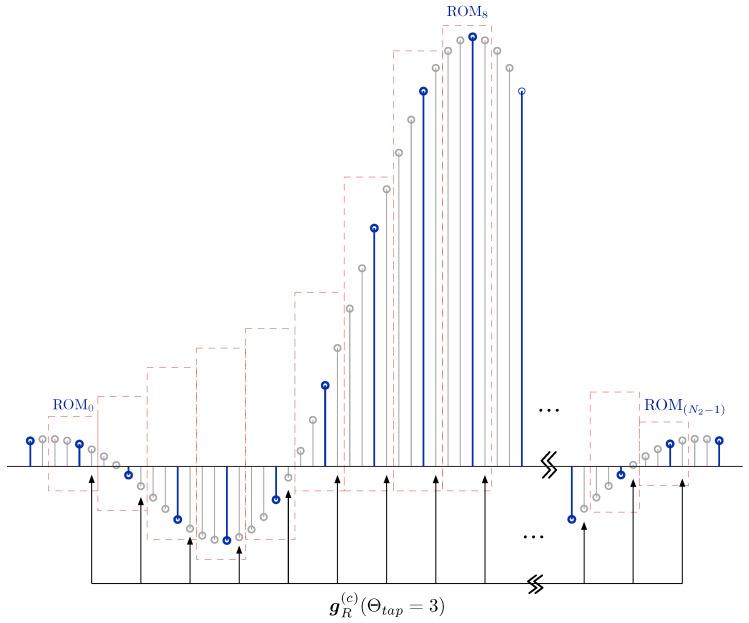
The extended SRRC pulse in discrete time domain with K=4, $S_{p}=4$ and $S_{l}=4$.

From the analysis above, the value of μ is limited to a fixed interval (i.e., μ∈[−0.5,0.5)), namely the timing error is at range from the left middle point of one ADC sampling point to the right middle point of the same point. To achieve timing phase error compensation accurately between every two ADC sampling points according to (4), it is necessary to generate a long series of matched filter coefficients in advance. That is, *K* points between every two samples of the matched filter should be inserted, where *K* is called filter oversampling multiple value and it usually takes as an integer multiple of 2 for implementation. According to [35,36], we get the extended MMF coefficients sequence $g_{p}$. Figure 5 shows an example of a SRRC pulse $g_{p}$ with α=0.35, $S_{p}^{\prime }=S_{p}K=4K$, $S_{l}=4$ and K=4. In this figure, blue points form a normal SRRC pulse with oversampling rate $S_{p}=4$ and gray points, which quantity is associated with *K*, are theoretical points between every two adjacent blue points at uniform intervals.

The input value μ from controller module cannot be used directly to get the MMF coefficients, because it is not integer and it is not suitable to lookup coefficients in practice. For implementation, we introduce an index $\Theta_{tap}$ to extract $g_{R}^{\left(c\right)}$ from the extended sequence $g_{p}$,

(21) $$\begin{array}{cc} \Theta_{tap}\left(c\right) & =\frac{K}{2}+\left\lfloor \mu (c)K\right\rfloor \\ \; & =\left\lfloor \left(\frac{1}{2}+\mu \left(c\right)\right)K\right\rfloor . \end{array}$$

Since μ∈[−0.5,0.5), the index of the MMF coefficients $\Theta_{tap}\in \left[0,K-1\right]$. For parallel implementation, we split the coefficients $g_{p}$ into $N_{2}=S_{l}\times S_{p}+1$ sections with uniform interval *K* and these sections are stored in $N_{2}$ independent ROMs (namely, ${\mathrm{ROM}}_{i}$, $i=0,1,\cdots ,N_{2}-1$), respectively. As shown in Figure 5, each ROM is based on the samples corresponding to the matched filter of length $N_{2}$ without offset as the center (blue points), stores its $\left\lfloor K/2\right\rfloor$ points in the negative direction and $\left\lfloor K/2\right\rfloor -1$ points in the positive direction. Under this condition, its sliding range is [−K/2,K/2). The slid MMF coefficients $g_{R}^{\left(c\right)}$ is read from each ROMs by index $\Theta_{tap}$. Take $\Theta_{tap}=3$ as an example, $g_{R}^{\left(c\right)}$ is composed with the 3rd data stored in each ROM, as shown in Figure 5.

After reading the $g_{R}^{\left(c\right)}$ from ROMs, convolving sequences $x_{I}^{\left(c\right)}$ and $x_{Q}^{\left(c\right)}$ with it respectively to achieve timing phase error compensation and get output sequence $y^{\left(c\right)}$. In order to reduce resource consumption and improve operation efficiency, we use polyphase filtering technique to achieve matched filtering by dividing the convolution process into several smaller steps. According to the polyphase filtering theory, (13) can be rewritten as the form of *L* path parallel polyphase filtering form in *z*-transform domain:

(22) $$\begin{array}{cc} y_{I}^{\left(c\right)}\left[k\right] & =\sum_{i=0}^{k}g_{R}^{\left(c\right)}\left[i\right]x_{I}^{\left(c\right)}\left[k-i\right]+z^{-L}\left\{\sum_{i=k+1}^{L-1}g_{R}^{\left(c\right)}\left[i\right]x_{I}^{\left(c\right)}\left[L+k-i\right]\right\},\;0\leq k\leq L-2\\ y_{I}^{\left(c\right)}\left[L-1\right] & =\sum_{i=0}^{L-1}g_{R}^{\left(c\right)}\left[i\right]x_{I}^{\left(c\right)}\left[L-1-i\right]. \end{array}$$

Since the length of the filter is $N_{2}$, it means $g_{R}^{\left(c\right)}\left[N_{2}+1\right]∼g_{R}^{\left(c\right)}\left[L-1\right]$ are all equaling to 0, the equation can be further simplified to reduce the calculational complexity.

### 4.2. Data Cache Implementation

**Figure 6:**
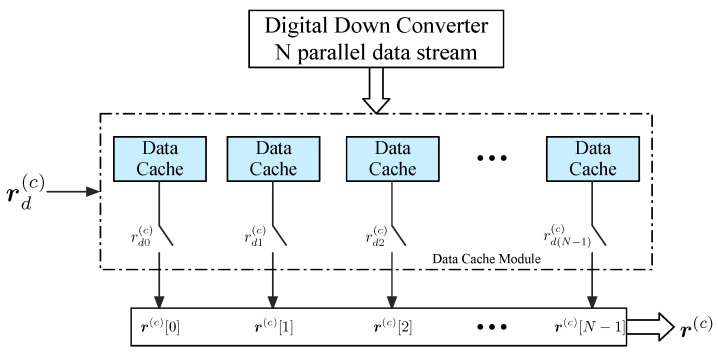
The control switch of the data cache.

As the timing delay accumulate, timing sampling point offset which causes timing phase error compensation by MMF mentioned above failure is inevitable. According to the analysis in Section 3.2, η is used to control data cache module, that is, the offset correction is achieved by reserving or deleting the sampling points. Under the *N*-path parallel architecture, data sampled by the ADC are stored in *N* parallel data caches. We introduce a control vector $r_{d}^{\left(c\right)}$ to determine the data reading behavior of data caches,

(23) $$r_{d}^{\left(c\right)}={\left[r_{d0}^{\left(c\right)},r_{d1}^{\left(c\right)},\;\cdots ,\;r_{d(N-1)}^{\left(c\right)}\right]}^{T}\in {\left\{0,1\right\}}^{N},$$

where $r_{di}^{\left(c\right)}$ is the *i*-th data cache’s control signal at *c*-th clock.As is shown in Figure 6, $r_{di}^{\left(c\right)}$ can be used as a switcher of the *q*-th cache. $r_{di}^{\left(c\right)}=1$ indicates that the *i*-th switcher is on so that *i*-th data cache is allowed to read data at the *c*-th clock. And when $r_{di}^{\left(c\right)}=0$, the switcher is off and there is no output from the *i*-th cache.

The value of the control vector $r_{d}^{\left(c\right)}$ is determined by the timing sampling point offset η(c). When η(c)=0, it means that there is no timing sampling point offset at the current moment and all data caches read data normally, that is, $r_{d}^{\left(c\right)}$ is a full 1 vector. When η(c)=−1, the ADC sampling clock is slower than the true symbol rate and one sampling point needs to be reserved. At this point, the *q*-th data cache does not read data and maintains the previous output,

(24) $$\left(r_{d}^{\left(c\right)}\leftarrow_{q}0\right)={\left[r_{d0}^{\left(c\right)},\;\cdots ,\;r_{d(q-1)}^{\left(c\right)},0,\;\cdots ,\;r_{d(N-1)}^{\left(c\right)}\right]}^{T},$$

where *q* is a decremental value with range $\left[0,N-1\right]$, i.e., q=q−1 when the next η(c)=−1 is coming.

When η(c)=1, the ADC sampling clock is faster than the true symbol rate and one sampling point needs to be deleted. Firstly, reading the data that need to be deleted from *p*-th data cache which will be temporarily stored without entering the timing recovery loop. *p* is an incremental value with range $\left[0,N-1\right]$, i.e., p=p+1 when the next η(c)=1 is coming. At this time, we introduce an enable signal C to control whether to discard the sequence $r^{\left(c\right)}$ read from data caches. When C=0, the output sequence $r^{\left(c\right)}$ is discarded, that means the current sequence $r^{\left(c\right)}$ will not enter into the timing recovery loop. The system suspends the current operation and waits for the next clock to come. Then, all *N* parallel data caches read data normally.

The algorithm proposed in this paper to achieve the reservation or deletion of sampling points of data caches in the timing recovery loop can be described as Algorithm 2. In FPGA implementation, due to the high clock stability achievable in current industrial applications, timing synchronization failure caused by clock accumulation can be neglected.
**Algorithm 2** Timing sampling point offset correction**Input:** The timing sampling point offset indicator η(c−1).  **Initialization:** Initialize the vector $r_{d}^{\left(c\right)}$(${\left[r_{di}^{\left(c\right)}\right]}_{i=0}^{N-1}=1$), q=N−1 and p=0.  1: **if**
η(c−1)=−1
**then**  2:     q=mod(q−1,N) and ${\left[r_{di}^{\left(c\right)}\right]}_{i=0}^{N-1}=1$  3:     $\left(r_{d}^{\left(c\right)}\leftarrow_{q}0\right)$    C=1  4: **else if**
η(c−1)=1
**then**  5:     p=mod(p+1,N) and ${\left[r_{di}^{\left(c\right)}\right]}_{i=0}^{N-1}=0$  6:     $\left(r_{d}^{\left(c\right)}\leftarrow_{p}1\right)$    C=0  7: **else**  8:     ${\left[r_{di}^{\left(c\right)}\right]}_{i=0}^{N-1}=1$  9:     C=1  10: **end if**  11: Read data sequence $r^{\left(c\right)}$ from data caches (DCs).  12: $r^{\left(c\right)}\left[m\right]$ = $\mathrm{DCs}(r_{d}^{\left(c\right)}\left[m\right])$.  13: Rearrange the output data which read from data caches to get the correct output sequence $r^{\left(c\right)}$.  14: Determine whether the output data $r^{\left(c\right)}$ is input to the timing recovery loop.  **Output:** The data sequence $r^{\left(c\right)}$ that read from data cache module.

### 4.3. Filter Oversampling Multiple Value

The value of *K* is important to the accuracy of timing phase error compensation, which can severely affect the performance of the loop. To analyze clearly, we introduce a value $v_{k}$,

(25) $$\begin{array}{cc} v_{k} & =\frac{\left\lfloor K\times \mu \right\rfloor }{K}-\mu , \end{array}$$

which represents the difference between the compensation value of the timing phase error through the MMF module and the actual timing phase output. It can be seen from (25) that the larger the value of *K*, the higher the timing recovery accuracy of the loop. However, in actual communication system, K→∞ is impractical. According to the requirements of timing recovery performance, selecting an appropriate *K* value is very important. It should be noted that in FPGA implementation, the larger the the value of *K*, the larger the storage resources consumption. We use numerical simulation to determine how to select an appropriate *K* value, which can save resources to the greatest extent while ensuring the timing recovery performance.

## 5. Simulation and Hardware Implementation

This section aims to verify the feasibility of the proposed timing recovery loop. Sampling deviations are introduced in numerical simulations to emulate the timing errors encountered in practical communication systems, thereby evaluating the timing recovery performance of the proposed loop. To further validate the algorithm’s feasibility under real-world operating conditions, a hardware platform for terahertz communication systems is established to conduct experimental tests on the proposed timing recovery loop.

### 5.1. Numerical Simulation Results

**Figure 7:**
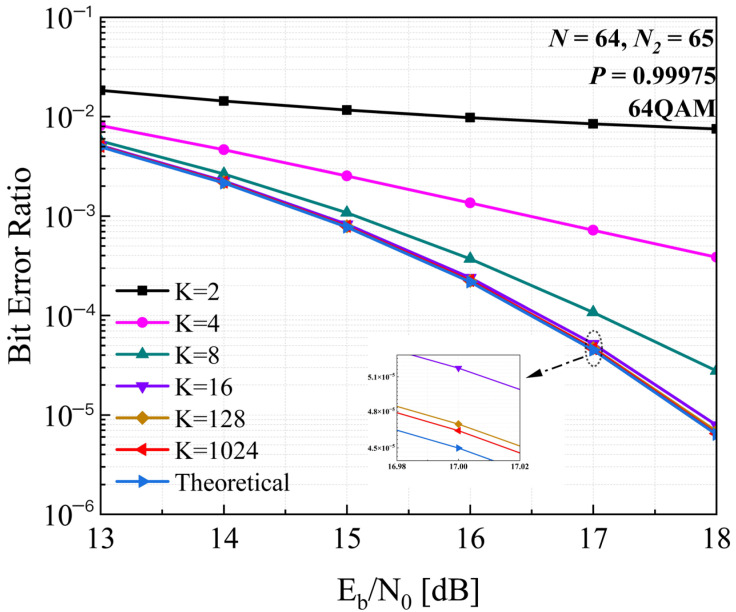
The BER curve for different *K* under P=0.99975.

This section presents numerical simulations of the proposed timing recovery loop based on MMF compensation. The feasibility of the loop is evaluated using two key indicators: the clarity of the constellation diagram after timing recovery, and the proximity of the system’s BER to the theoretical BER—with the deviation between the post-recovery BER and the theoretical BER required to fall within an acceptable range. As detailed in Section 4.3, a suitable value of parameter *K* is determined through loop performance simulation before FPGA implementation. Figure 7 illustrates the BER curves corresponding to different *K* values under a timing sampling deviation of P=0.99975, where *P* denotes the timing sampling deviation, that is, resampling the transmitter sequence at *P* times of the original sampling rate.

When K≤4, the BER curve decreases slowly, and the performance gap tends to widen as $E_{b}/N_{0}$ increases. When K≥16, the BER curve tends to align with the theoretical BER curve, and with the increase of *K*, the performance loss is less than 0.1 dB compared to the theoretical BER curve. Thus, we choose K=32 for both loop performance simulation and hardware implementation. The parameters of this architecture is shown in Table 1.

**Figure 8:**
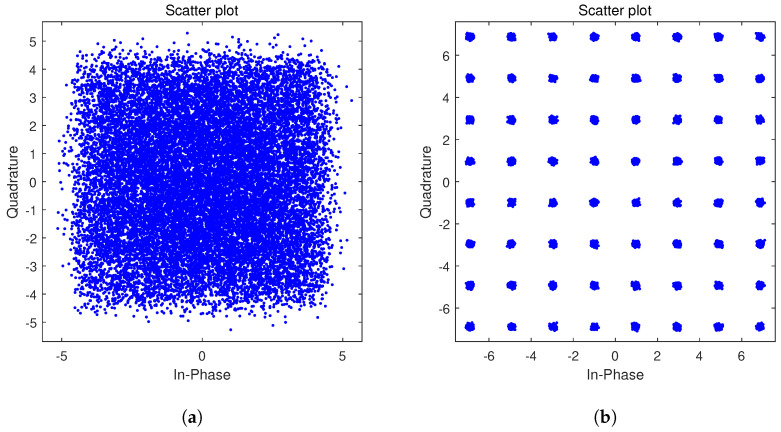
The constellation diagrams before (**a**) and after (**b**) timing recovery loop.

**Figure 9:**
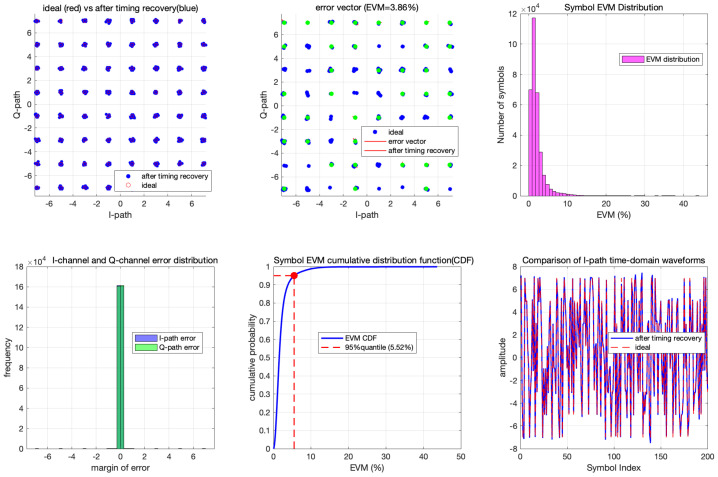
The EVM performance after loop.

**Figure 10:**
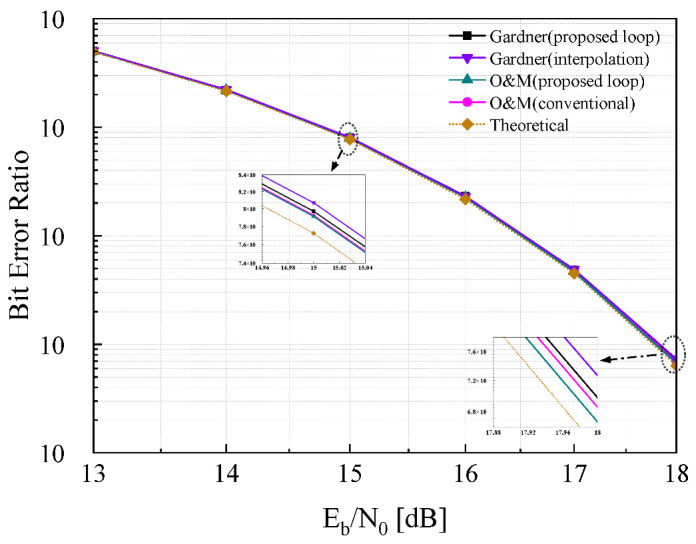
The BER curve of the loop.

**Figure 11:**
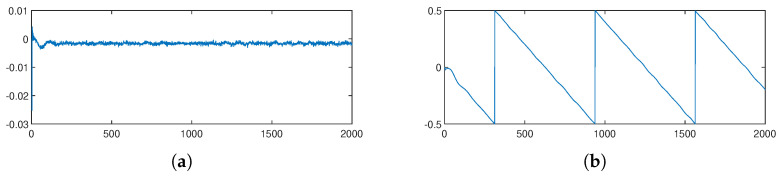
Parallel timing recovery system output parameters. (**a**) is the smoothed timing error estimated $\mu_{l}$ of the system. (**b**) is the the timing phase out μ.

To comprehensively characterize the performance of the proposed timing recovery loop, numerical simulation results are presented in this part. Figure 8 illustrates a comparison of the constellation diagrams before and after the operation of the timing recovery loop: Figure 8a shows the constellation diagram prior to loop processing, while Figure 8b depicts the constellation diagram after loop optimization. A clearly distinguishable 64QAM constellation pattern is observed in Figure 8b, demonstrating the effective timing recovery capability of the proposed loop. And the EVM performance is shown in Figure 9, after timing recovery, the average EVM of the system is 3.86%, and most symbols have EVM values concentrated in the range of 0 to 4%. To further validate the loop’s performance under noisy conditions, additive white Gaussian noise (AWGN) is introduced into the received sequence, and BER curves are obtained over an $E_{b}/N_{0}$ range of 13 dB to 18 dB is given. Figure 10 presents the BER curves corresponding to different TED algorithms. It can be observed that the performance of the proposed timing recovery loop outperforms the algorithms reported in [25,28]. Figure 11a illustrates the smoothed timing error estimate $\mu_{l}$ of the system, and Figure 11b depicts the NCO output μ. As observed from the figures, the system output $\mu_{l}$ oscillates around the actual timing error value. Combined with the analysis results presented in Figure 8, Figure 9, Figure 10 and Figure 11, we can draw a conclusion that the proposed joint timing recovery loop exhibits efficient operational performance in practical scenarios.

### 5.2. FPGA Implementation and Experimental Results

To verify the feasibility and practicality of the proposed time-domain joint algorithm, a terahertz high-speed communication transceiver verification platform was established, and corresponding hardware tests were conducted. The main implementation parameters are listed in Table 1, with the timing sampling deviation introduced by the actual communication system. Specifically, the filter length was selected as $N_{2}=33$, which balances loop performance and hardware resource consumption effectively.

**Figure 12:**
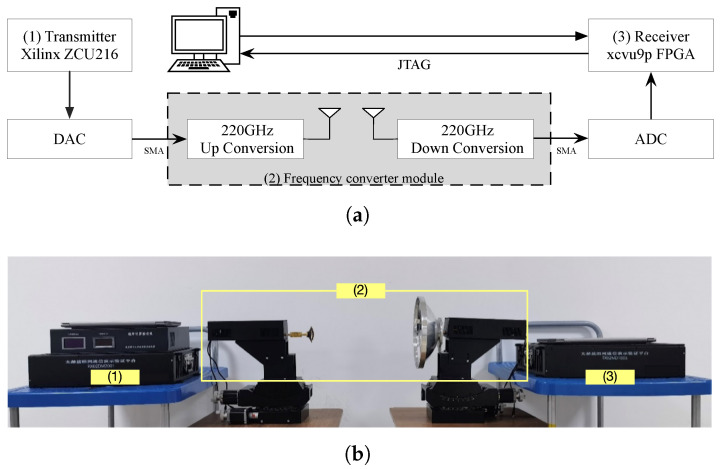
The hardware verification platform of the timing recovery loop. (**a**) is the topology of the platform. (**b**) is the photograph of platform.

The topology and physical photograph of the hardware verification platform are illustrated in Figure 12a,b respectively. Xilinx Zynq UltraScale+ RFSoC ZCU216 (ZU49DR) Evaluation Kit (marked as (1) in Figure 12b) was employed to generate a 64QAM-modulated signal with a data rate of 15 Gbps (2.5 Gsps) at an intermediate frequency (IF) of 2.5 GHz. The generated digital sequence from FPGA is converted into analog signal by one of DAC channels of XM655 16T16R Breakout Add-on Card and then mixed with radio frequency (RF) of 220 GHz [37,38,39] by subharmonic mixer (marked as (2) in Figure 12b), which is driven by 12.222 GHz reference clock from signal source. Finally, the transmitting signal propagates through a THz antenna.

At the receiver, the 220 GHz RF signal captured by the THz antenna was amplified by a low-noise amplifier (LNA) before being downconverted to an IF signal via another subharmonic mixer. The IF signal was then digitized by an ADC channel of the TI ADC12DJ5200RF EVM board at a sampling rate of 10 Gsps. A Xilinx UltraScale+ VCU118 (XCVU9P) Evaluation Kit (marked as (3) in Figure 12b) was utilized to implement the full receiver signal processing chain, including the proposed timing recovery loop, equalization, carrier synchronization, and 64QAM demodulation. The full-flow receiver data (from ADC input to demodulated output) was recorded to verify the end-to-end performance. The constellation diagram and BER after each processing stage were monitored to confirm the effectiveness of the proposed timing recovery algorithm in the actual receiver chain.

**Figure 13:**
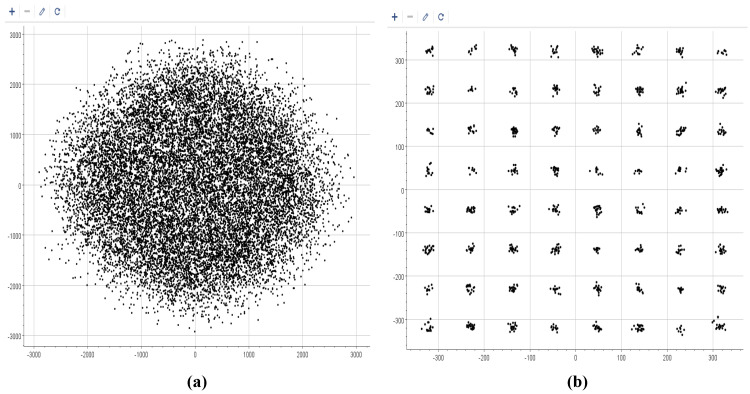
The constellation diagram before (**a**) and after (**b**) the timing recovery process of the received signal.

Firstly, a system direct-connection test was conducted, where the only timing deviation introduced into the system originated from the ADC sampling instants. This test scenario focuses on verifying the core performance of the proposed timing recovery loop under a simplified yet practical non-ideal condition. The constellation diagrams after timing recovery were displayed in ChipScope software on a PC via the JTAG interface. Figure 13a presents the constellation diagram of the received signal before timing recovery, while Figure 13b shows the constellation diagram after the proposed timing recovery loop is enabled. It can be observed that the proposed loop achieves excellent performance in mitigating ADC-induced sampling deviations, as evidenced by the fully distinguishable 64QAM constellation.

**Figure 14:**
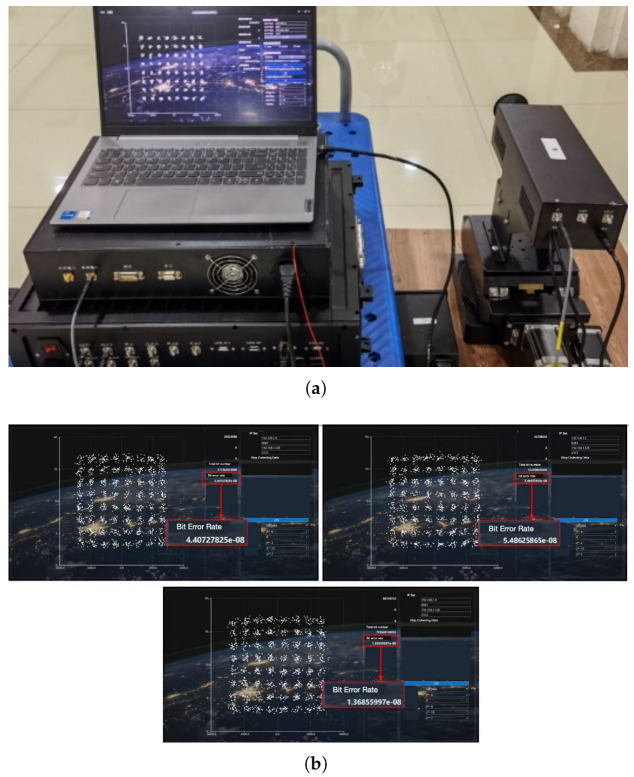
(**a**) is the topology of the platform. (**b**) is the photograph of platform.

Subsequently, a long-distance THz communication field test was carried out to further validate the loop’s performance in practical application scenarios. In this test, the proposed timing recovery loop was integrated as a key stage in the receiver signal processing chain to jointly complete system demodulation. The received constellation diagrams and BER of the system were statistically analyzed through three independent test runs, with the measured results presented in Figure 14. Figure 14a shows the physical photograph of the receiver hardware platform deployed in the field test, while Figure 14b depicts the three sets of statistical BER results from the independent tests. The average BER across the three measurements is $3.75\times 10^{-8}$, and the post-demodulation constellation diagram exhibits clear clustering of 64QAM symbol points. These results fully demonstrate the superior performance, excellent stability, and reliable practicality of the proposed timing recovery loop in real long-distance THz communication systems.

Table 2 presents the FPGA resource utilization results and comparison with other studies under the same environment with geometric mapping. It can be seen our architecture shows a consistent advantage in LUT and BRAM utilization. Compared with existing timing recovery loops, this loop avoids a large number of FFT/IFFT operations or interpolation, reduces the computational complexity of the system. The advantage of the proposed loop is even more significant as the number of parallel paths *N* increases.

## 6. Conclusions

This paper proposes a joint parallel timing recovery loop based on a time-domain feedback architecture, with its performance comprehensively validated through numerical simulations and hardware experiments. Under the condition of an AWGN channel with a timing sampling deviation factor P=0.99975 introduced, the proposed loop was compared with conventional parallel loops, and the simulation results demonstrate that its BER aligns well with the theoretical curve, incurring a performance loss of less than 0.1 dB with 64QAM modulation. For hardware validation, the loop was successfully deployed on the Xilinx UltraScale+ VCU118 (XCVU9P) Evaluation Kit and integrated into a 220 GHz communication system. System-level tests confirm its capability to support real-time demodulation of 15 Gbps 64QAM signals. As a key stage in the receiver, it enables the system to achieve an average BER of $3.75\times 10^{-8}$, verifying the loop’s excellent stability and practicality in supporting end-to-end system performance. Notably, the architecture avoids extensive FFT/IFFT operations and interpolation, significantly reducing hardware resources. However, higher synchronization precision requires storing more MMF coefficients, and its performance is achieved at the cost of ROM resources. Combined with its inherent high parallelism, the proposed loop effectively addresses the resource constraints of on-board systems and meets the evolving demands of next-generation large-bandwidth, high-speed THz communication.

## Figures and Tables

**Table 1:** Basic parameters of timing recovery loop.

System Parameters	Variable	Value
Parallel paths	*N*	64
Modulation order	*M*-QAM	64-QAM
SRRC filter length	$N_{2}$	65
Up-sampling ratio	$S_{p}$	4
Symbol rate	$R_{s}$ (Gsps)	2.5 Gsps
Timing sampling deviation	*P*	0.99975
TED	/	O&M
Filter oversampling multiple value	*K*	32

**Table 2:** Resource Occupation Comparison.

Logic Utilization	Proposed	In [5] Proposed	In [28] Proposed
DSPs	2241	1060	837
Slice LUTs	67,806	81,000	74,172
Slice Registers	66,061	46,900	104,600
BRAM	16	153	380

Table exists mapping estimation.

## Data Availability

Data are contained within the article.

## Abbreviations

The following abbreviations are used in this manuscript:

THz	Terahertz
MMF	Modified matched filter
TED	Timing error detector
DCs	Data caches
BER	Bit error rate
FPGA	Field-programmable gate array
IF	Intermediate frequency
ADC	Analog to Digital Converter
NCO	Numerical control oscillator
SRRC	Square-root-raised-cosine
LF	Loop filter
